# Preference‐based patient participation in intermediate care: Translation, validation and piloting of the 4Ps in Norway

**DOI:** 10.1111/hex.13899

**Published:** 2023-11-07

**Authors:** Linda A. H. Kvæl, Astrid Bergland, Ann C. Eldh

**Affiliations:** ^1^ Department of Rehabilitation Science and Health Technology Faculty of Health Sciences, Oslo Metropolitan University Oslo Norway; ^2^ Norwegian Social Research—NOVA Oslo Metropolitan University Oslo Norway; ^3^ Department of Health, Medicine and Caring Sciences Faculty of Medicine and Health Sciences, Linköping University Linköping Sweden; ^4^ Department of Public Health and Caring Sciences Uppsala University Uppsala Sweden

**Keywords:** careful translation, content validity, intermediate care, patient participation, person‐centred care, the 4Ps

## Abstract

**Introduction:**

The implementation and evaluation of patient participation to obtain high‐quality transitional care for older people is an international priority. Intermediate care (IC) services are regarded as an important part of the patient's pathway from the specialist to the primary care levels, bridging the gap between the hospital and the home. Patients may experience varying capacities and conditions for patient participation. Yet, few tools for evaluating patients' preferences for patient participation within IC services are at hand. Accordingly, further knowledge is needed to understand and scaffold processes for patient participation in IC. Therefore, the aim of this project was to translate, validate and pilot test the Patient Preferences for Patient Participation (the 4Ps) with patients in IC services in Norway.

**Methods:**

This project comprised two phases: (1) a careful translation and cultural adaptation process, followed by a content validity trial among 15 patients and staff in Norwegian IC and (2) a cross‐sectional survey of the instrument with 60 patients admitted to IC.

**Results:**

The translation between Swedish and Norwegian required no conceptual or contextual adaptations. The subsequent cross‐sectional study, designed as a dialogue between the patients and staff, revealed that only 50% of the participants received a sufficient level of patient participation based on their preferences, mostly indicating that patients were receiving less‐than‐preferred conditions for engaging in their health and healthcare issues.

**Conclusion:**

The 4Ps instrument was deemed suitable for measuring patient participation based on patient preferences in the IC context and was feasible for both healthcare professionals and patients to complete in an interview when arriving at and leaving services. This may support person‐centred communication and collaboration, calling for further research on what facilitates patient participation and the implementation of person‐centred services for patients in IC.

**Patient or Public Contribution:**

First, the current paper is part of the IPIC study (i.e., the implementation of patient participation in IC). Influenced by a James Lind Alliance process, the study addresses research uncertainties identified by patients, next of kin, staff and researchers in the cocreation process. Second, cognitive interviewing was conducted with 15 representatives of the target population: seven patients receiving IC services, one home‐dwelling previous IC patient (altogether four women and four men, most of them 80 years or older) and seven healthcare staff working in IC services. The interviews determined the relevance, comprehensiveness and clarity of the 4Ps. Finally, 60 patients admitted to IC took part in the cross‐sectional study.

## 1 BACKGROUND

Healthcare laws and policies around the world include the right of patients to engage in their health, care and treatment—that is ‘patient participation’.^1^ Implementing and evaluating patient participation to obtain high‐quality transitional care for older people is an international priority.^2^ Individual patient participation encompasses the entitlements and possibilities for patients to partake in decision‐making regarding their healthcare, achieved through a dialogue that incorporates the patient's preferences, resources, and a fusion of the patient's experiential knowledge and professional expertise.^3^ The use of patient‐reported outcomes in healthcare is progressively growing due to their alignment with health policies.^4^ Therefore, patient‐reported outcomes must be valid for the given context and the patient group, depending on whether the respondents understand the questions and response options, whether they are relevant to the concept being measured and whether the important aspects of that concept are comprehensively captured.^5^ This paper focuses on measurements of patients' participation in intermediate care (IC), addressing their preferences for and experiences with conditions for their engagement in health and healthcare issues.

IC services are an important part of the patient pathway from the specialist to the primary care levels and aim to bridge the gap between the hospital and the home^6^, ^7^, ^8^ to deliver rehabilitation posthospitalisation^9^ or to prevent hospital (re)admission.^10^ Services are community based and account for a limited portion of the healthcare process,^11^ calling for patient engagement to aid in individuals' well‐being and progress. Family meetings are a cornerstone of IC and effectively use an important arena to facilitate patient participation.^12^, ^13^ Family meetings incorporate a sit‐down with the patient, their relatives, the IC team and the district coordinator to agree upon goals and interventions during their rehabilitation, the length of the stay and follow‐up services after discharge.

While patient participation is conceptualised in a variety of ways, more recent evidence emphasises the recognition of patients' needs, goals and resources to obtain quality healthcare.^1^, ^14^ To conform with person‐centred values, healthcare professionals (HCPs) should invite patients and their relatives to actively be involved in, for example, shared decision‐making based on their own preferences.^15^, ^16^ Despite decades of advocacy for such norms, such person‐centredness is not yet mainstream practice.^17^, ^18^, ^19^, ^20^

Patient participation implies core elements, such as the patient being recognised as an individual who can understand what the illness means for their everyday life, considering their values and perspectives and the sharing of power and responsibility.^21^ Shared decision‐making has been suggested as a way to involve patients in planning and treatment decisions based on their priorities.^22^ Hence, there is a need to better understand how to capture and address patient participation; structured and accessible evaluations can advance patients' participation in IC services. Earlier studies indicate a great potential to deliver services in a more person‐centred way.^20^, ^23^, ^24^, ^25^ Yet, few tools for person‐centred patient participation within IC services are at hand.^13^, ^20^ Accordingly, further knowledge is needed to understand and scaffold processes for patient participation in IC.^7^, ^24^

Today, HCPs may need support to capture and implement more person‐centred patient participation in daily clinical routines.^23^, ^26^ Any broad concept incorporating attributes such as patient participation^20^ calls for tools enabling older people to denote their preferences for patient participation. Since these may vary, such tools must be valid yet easily administered.^23^, ^27^ A systematic review reveals a few valid evaluation tools that emphasise patient participation as conceptualised by patients. There are even fewer considering patient participation exclusively^28^—and there is none in Norwegian.

However, a more recent instrument was validated for Swedish healthcare: the Patient Preferences for Patient Participation, or the 4Ps.^29^ This allows individuals to consider and share their preferences for and experiences with patient participation. The 4Ps tool^30^ provides not only evaluation opportunities but also conditions for a dialogue on preferences when initiating a healthcare interaction.^30^, ^31^ The 4Ps incorporate attributes corresponding to semantic, legislative and conceptual aspects of patient participation, yet also connote overarching care goals like person‐centred care, self‐management and shared decision‐making.^32^, ^33^ Hence, the 4Ps is a patient‐reported experience measure (PREM) for patient participation. The tool consists of two sections: one for a patient's preferences for participation and another for that individual's experience with participation in their health and healthcare. It sports 12 items, including what has been defined as patient participation with concept analyses, including semantics, healthcare terminologies and classifications and, most importantly, patient experiences.^29^ All attributes are presented in Table 1.

**Table 1:** The 12 items conceptualising participation in the 4Ps.

No.	Items of the 4P tool
1	Being listened to (by healthcare staff).
2	My experiences being recognised.
3	Having reciprocal communication.
4	Sharing one's symptoms or issues.
5	Having explanations of my symptoms.
6	Being informed of what is being done (for me).
7	Learning of plans (for me).
8	Taking part in planning.
9	Phrasing personal goals.
10	Learning to manage symptoms.
11	Managing treatment, myself.
12	Managing self‐care.

Abbreviation: 4P, Patient Preferences for Patient Participation.

For each of the two sections, the 12 items are echoed, but in a different tense: First, the patient illustrates his/her preferences for patient participation as ‘to me, to facilitate my patient participation’, [each item] it is ‘not important’, ‘somewhat important’, ‘very important’ or ‘crucial’. Subsequently, the patient demonstrates to what extent he/she has experienced patient participation. Using the same 12 items, the patient indicates (in the past tense) whether the conditions [for each item] were facilitated: ‘not at all’, ‘to some extent’, ‘to a large extent’ or ‘entirely’.^29^ The two sections are then compared in a clinical dialogue, by a researcher or by a quality management representative to evaluate whether there was a fit between the individuals' preferences for and experiences with patient participation.

With growing demand for a more person‐centred healthcare system in general and IC in particular, this study addressed the lack of valid tools measuring patient participation in the Norwegian IC context to meet the need for patient‐reported outcomes that particularly recognise patients with need for further care after hospitalisation.^34^

### 1.1 Aims of the study

The aim of this study was to translate, validate and pilot test the 4Ps with patients in IC services in Norway.

## 2 METHODS

### 2.1 Design

This project comprised two phases: (1) a translation and cultural adaption process, followed by a content validity trial in Norwegian IC^35^, ^36^, ^37^ and (2) a cross‐sectional survey of the instrument with patients admitted to IC.

### 2.2 Phase 1: Translation and content validity process

For careful translation, we followed widely recognised recommendations,^35^, ^36^, ^38^ embracing a multistep process as an integral part of content validity.^36^ ‘Content validity’ implies ‘the degree to which the content of an instrument is an adequate reflection of the construct to be measured’,^39^ ^,p.743^ and is the most important measurement property for PREMS. It examines the relevance, comprehensiveness and comprehensibility of the phenomenon measured.^37^

To commence the project, a research team was formed consisting of three researchers: a postdoctoral fellow with prior studies in IC and two professors from Norway and Sweden, respectively. Both had experience with tool translations and cross‐cultural adaptations, and one had developed and trialled the 4Ps in Sweden.

#### 2.2.1 Forward translation and reconciliation

The 4Ps tool was primarily translated independently from Swedish (a language with many similarities to Norwegian) into Norwegian by the two researchers on the team. Norwegian is their mother tongue; both have comprehensive knowledge of the patient participation construct. Particular attention was given to the potential to capture and phrase language and cultural dimensions while being linguistically as accurate as possible.^38^ The two translations were compared to finalise a single translated version of the Norwegian 4Ps.^36^

#### 2.2.2 Backward translation and harmonisation

The Norwegian version of the 4Ps instrument was then backward translated into Swedish by two independent bilingual translators blinded to the original version. Swedish was the native language of these two translators, and each translation included comments and notes. Furthermore, the two back‐translated versions of the instrument were compared with the original and all translated versions in a harmonisation meeting.^36^ An agreed‐upon version of the 4Ps Norwegian instrument was obtained with the developer of the original version.

#### 2.2.3 Cognitive debriefing

Content validity was assessed using cognitive interviewing following the ‘Think Aloud’ method.^40^ Cognitive interviewing was conducted with 15 representatives of the target population: seven patients receiving IC services, one home‐dwelling previous IC patient (altogether four women and four men, most of them aged 80 or older) and seven healthcare staff working in IC services (one registered nurse, one nursing assistant, one physical therapist, one quality manager and three occupational therapists).

The interviews were individual and face‐to‐face and evaluated the instructions, the response format, the 4P items and the response alternatives.^38^, ^41^ During the interviews, the informants were asked to fill out the 4Ps while thinking aloud and addressing the relevance, comprehensiveness and clarity of the 4Ps.^40^ An interview guide was developed in line with COnsensus‐based Standards for the selection of health Measurement INstruments,^37^ that is COSMIN's criteria for good content validity (File S1). The researcher subsequently wrote down the informants' feedback, and after a thematic analysis of the findings,^42^ a discussion among the researchers completed a third and final version.

### 2.3 Phase 2: Piloting the 4Ps instrument

Based on feedback from the cognitive debriefing in Phase 1, the data collection for the cross‐sectional study with a repeated measure was set up as a dialogue between the patients and their HCPs.^29^

#### 2.3.1 Setting

The piloting of the 4Ps instrument was carried out in four IC institutions in two cities in Norway. The four IC institutions had 68–142 beds and provided short‐term rehabilitation bridging the pathway from the hospital to the home to enable older people to maintain their independence following a period of illness. Patients in IC are mainly older people in need of further care, but younger patients with complex health issues may also use the services. The length of stay in the IC services are time‐limited, for example, 2–3 weeks.^11^

#### 2.3.2 Participants

The study's participants were patients (54–99 years) admitted to IC services as part of their pathway; all were recruited through a consecutive sampling method. The criteria for inclusion in the study were: patients transferred to IC due to chronic/long‐term disease or frailty (i.e., more than 6 months), dependent on activities of daily living (ADL) on admission day but wanting to pursue ageing in place (i.e., to be discharged to their home). Patients with severe cognitive impairments, inadequate Norwegian language skills or aphasia were not included.

#### 2.3.3 Data collection

Two HCPs from each IC institution handled the data collection. They identified suitable patients and collected data with consenting patients. To ensure uniformity and to standardise their interactions, a protocol was developed by the first author and provided at each site in a 1‐h training session. This protocol (available in full in File S2) was consistently used for data collection throughout the entire project. Altogether, eight HCPs (four physical therapists and four occupational therapists) collected data between February 2022 and February 2023. None of them collected data for the patients whom they treated. According to the protocol, the first section of the 4Ps, that is, the patient's preferences for patient participation, was completed within the first few days after the patient's admission. The second section, that is, the patient's experience with patient participation, was completed during any of the final few days before the patient was discharged to their home. Demographic data were obtained and registered at either of these data collection points, including age, sex, level of education and marital status. Furthermore, details were obtained from the patient's medical record regarding the number of medical diagnoses, the need for home care services and multimorbidity.

#### 2.3.4 Data analysis

**Figure 1:**
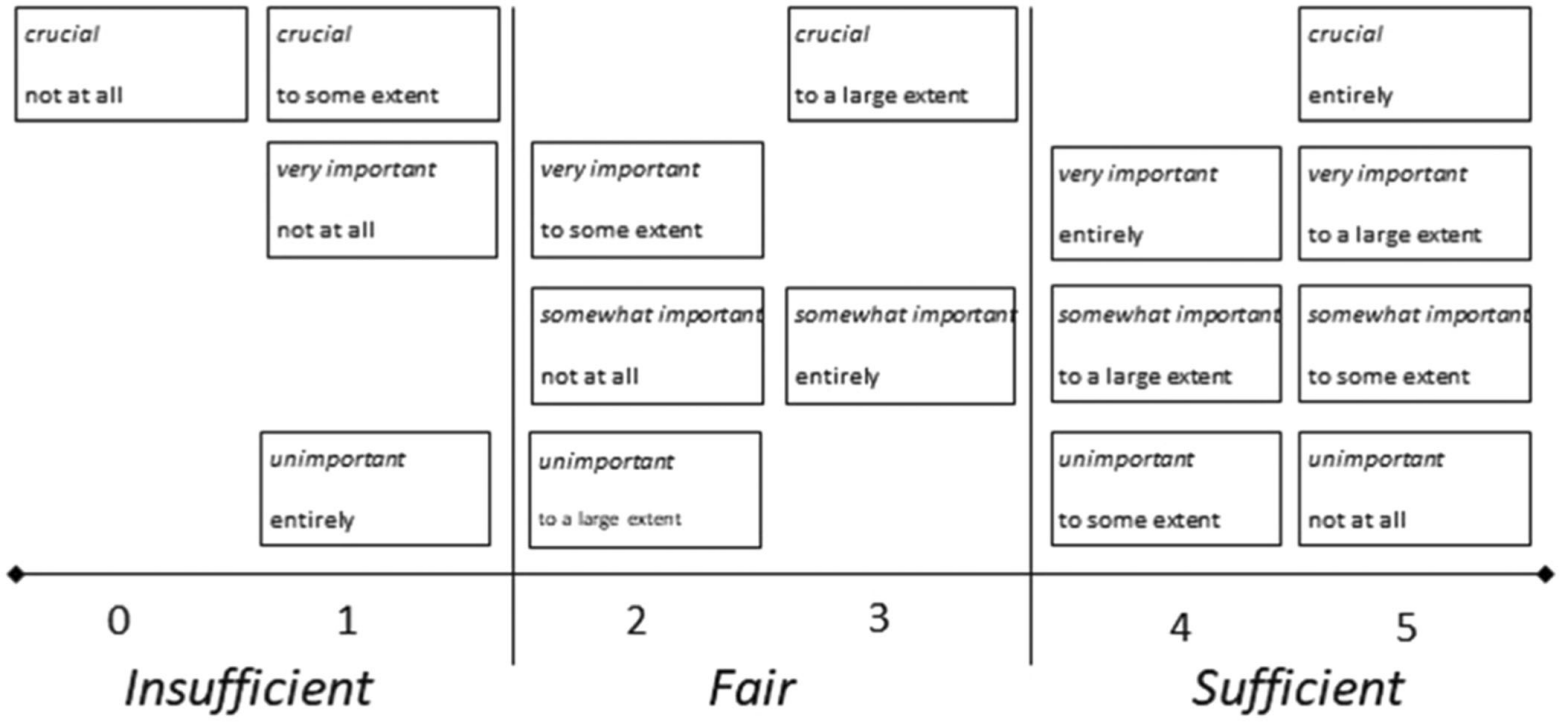
Ranks and levels of the matches and mismatches between patient preferences (*italics*) and patient experiences (roman). First published by Eldh et al.^31^

Statistical analyses were conducted using IBM Statistical Package for Social Sciences Version 27 software. With the 4Ps representing ordinal data, descriptive statistics were primarily gathered. However, to identify the degree of fit between the patients' preferences and experiences, six grades of potential matches were employed. These convey whether patients' preferences are reflected by their experiences,^30^, ^31^ as depicted by Figure 1.

Participant characteristics are summarised using descriptive statistics, presenting percentages and proportions for categorical variables, and means with standard deviation for interval data. Statistical differences between groups were assessed using the Mann–Whitney *U* test for nonnormality distributed interval data and the *χ* ^2^ test for categorical data. All statistical analyses were conducted with a significance level set at *p* < .05.

### 2.4 Ethics

This study received approval from the Regional Committees for Medical and Health Research Ethics (No. 107392) and was also presented to the Norwegian Centre for Research Data (No. 641210). After receiving both oral and written information, all subjects provided informed consent. They were further informed that they could withdraw from the study at any time without consequences. As part of the 1‐h data collection training in Phase 2, the data collectors for each site were instructed to apply strict guidelines for secure data storage until it could be handed to the research team. The researcher picked up the data consecutively and securely stored it on Services for Sensitive Data (TSD), a platform specifically designed for collecting and storing sensitive data in compliance with Norway's Privacy and Electronic Communication Directive.

## 3 RESULTS

The results will be presented in concordance with the two study phases: (1) the results of translation and cognitive interviewing for content validity and (2) the results of the cross‐sectional study based on 60 participants, in which the 4Ps were piloted in the IC context.

### 3.1 Phase 1: Translation and content validity

Overall, agreement was easily made, and neither the translators nor the project group members had to compromise. During the forward translation, small discrepancies in meaning were detected, such as ‘patient involvement’ versus ‘patient participation’, and the use of synonyms, such as ‘healthcare system’ versus ‘healthcare services’. Similarly, the back‐translated versions were close to the original. In the original version, the word ‘entirely’ was the fourth response alternative in Section 2, whereas the backward translators suggested ‘always’ and ‘completely’. However, a consensus was reached during the harmonisation meeting with the developer of the instrument.

The respondents interviewed found the 4Ps relevant, understandable and user friendly. Furthermore, the instructions were easy to understand, and the layout of the 4Ps was logical. Considering the level of patient frailty within IC services, it was considered important to attune the data collection in a dialogue between the patient and an HCP rather than the patient's completing it as a self‐report. Accordingly, in Phase 2, the first section of the 4Ps (on the patient's preferences for participation) used a structured interview based on the 4Ps within the first few days after a patient was admitted to the IC institution. The second section, on their experiences with patient participation, was completed in a second dialogue during the final days of their IC stay.

During the cognitive interviews, the phrase ‘having conditions for [attribute of patient participation]’ was considered novel, unfamiliar and somewhat difficult to grasp. The more casual word ‘opportunity’ was suggested in more everyday Norwegian. The research team found that, conceptually, ‘opportunity’ has a different connotation, indicating an option or opening for something. This would indicate that someone else has procured an opportunity, restricting the anticipated mutuality of participation (with its core connotation of sharing). Rather, ‘having conditions’ demonstrates that there has been an arena for something to occur, including the individual's needs, resources and efforts and the forms of negotiation provided in the healthcare interactions. Hence, the original intent was kept in the Norwegian version.

In addition, some respondents suggested changing the examples in Items 11 and 12 to better contextualise the 4Ps. Rather than ‘for example, manage my medication’, IC would be illustrated by ‘compliance with exercise’. Moreover, ‘being independent in my ADL’ would be more relevant than ‘adapting my diet’. Yet, the primary purpose of these examples was not to match the context but to reveal the difference between the prescribed treatment and self‐care. Thus, the examples were kept, given that any altered examples would limit the potential for comparisons. Yet, slight alterations were made, with core content solicited in three attributes/items, ensuring that the wording for symptoms/issues was consistent between Swedish and Norwegian (and English). Likewise, a modification to the introduction ensured that the wording in Norwegian equalled the instruction to consider one's current healthcare contact when responding to the 4Ps. See File S3 for the Norwegian 4P version.

### 3.2 Phase 2: Piloting of the 4P instrument

In Phase 2, a cross‐sectional design was employed to examine the extent and variation of patient participation based on the patients' preferences. The 60 patients (see Table 2) admitted to IC responding to the 4P instrument had a mean age of 79.8 years, ranging from 54 to 99 years of age. Of these, 58% (*n* = 35) were women. Approximately three out of five had obtained higher education, and 72.4% lived alone. The whole patient sample had a mean of three medical diagnoses and the median length of the IC stay was 14 days (*n* = 35).

**Table 2:** Patient characteristics of the entire sample according to gender.

	Registered (*n*)	Whole sample (*n* = 60)	Range (min/max)	Male (*n* = 25)	Female (*n* = 35)	*p*‐ Value
Age in years, mean (SD)	58	79.8 (11.5)	54–99	76.2 (10.1)	82.5 (11.8)	.02*
Education, high (*n*) (%)	51	37 (72.5)		19 (79.2)	18 (66.7)	.32
Marital status, living alone (*n*) (%)	58	42 (72.4)		13 (54.2)	29 (85.3)	.01*
Number of diagnoses, mean (SD)	58	3 (1.7)	1–7	2.3 (1.1)	3.5 (1.9)	.03*
Comorbidity, two or more diagnoses (*n*) (%)	58	33 (56.9)		12 (48)	21 (63.6)	.23
Number of home care services, mean (SD)	59	1.5 (1.7)	0–7	0.9 (1.2)	1.9 (1.9)	.03*

*Note*: Gender (1 = male and 2 = female), age expressed in years, education (1 = low: primary and high school and 2 = high: college and university), marital status (1 = living alone and 2 = living with partner), diagnoses and home care services given in numbers and comorbidity: two or more chronic diagnoses (1 = no and 2 = yes). *p*‐Value implies a significance level based on the *χ* ^2^ test for categorical data and the Mann–Whitney *U* test for interval data.

Abbreviations: *n*, number of registered; SD, standard deviation.

* *p* < .05.

Furthermore, more than half of the respondents (*n* = 33) could be characterised as multimorbid, implying that they had two or more chronic/long‐term diagnoses, and about 60% received home care services. Overall, the women were significantly older than the men (*p* = .02), more often lived alone (*p* = .01), had additional diagnoses (*p* = .03) and more often needed home care services (*p* = .03).

**Figure 2:**
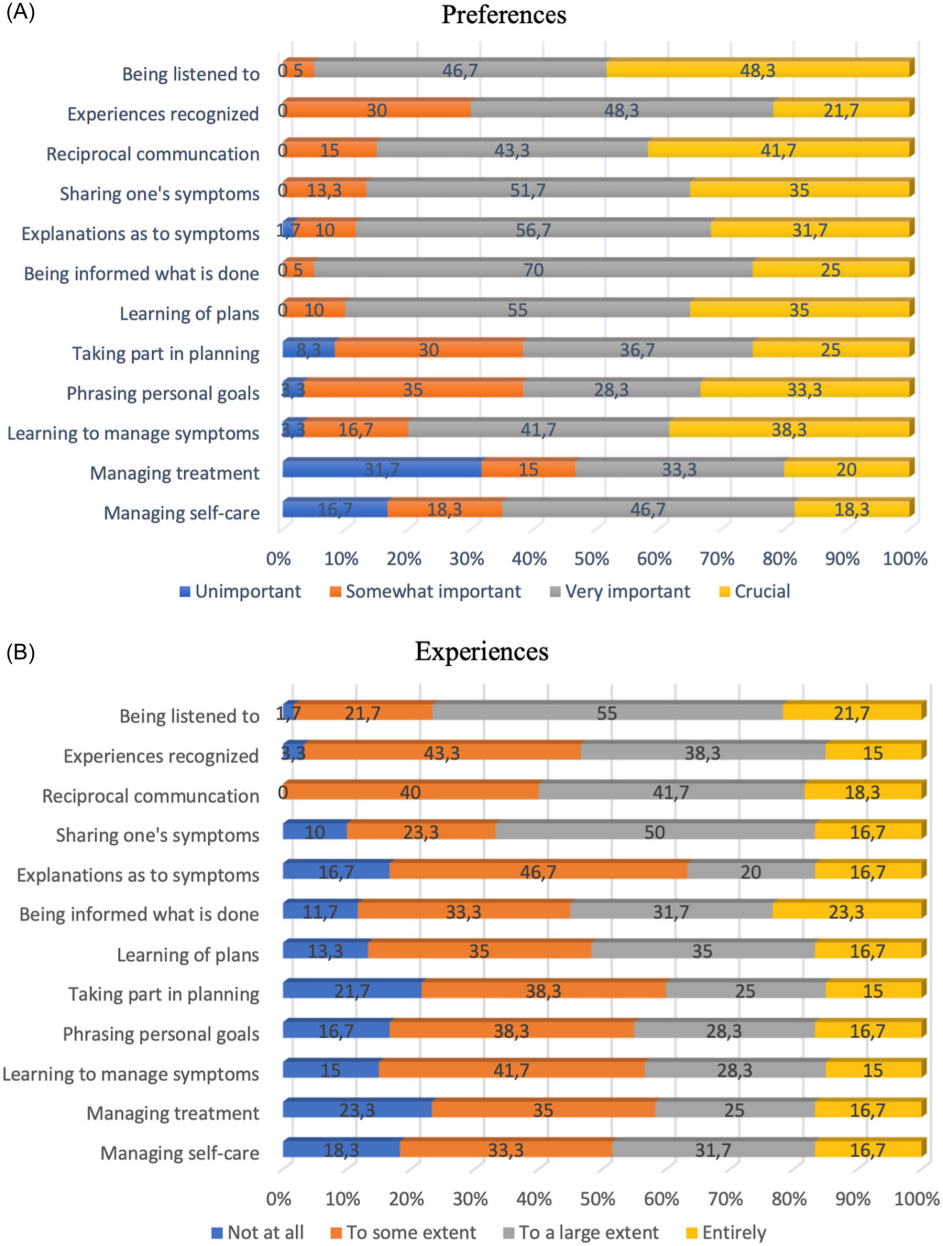
Patient preferences for patient participation (A) and patient experiences with patient participation (B) for each attribute of the 4P instrument. 4P, Patient Preferences for Patient Participation.

Regarding patient preferences for participation, the two items ‘being listened to’ (Item 1) and ‘having reciprocal communication’ (Item 3) were most often considered crucial for the patients' participation. Combining the second highest and highest alternatives (i.e., very important and crucial), two items trumped the patients' preferences with 95%: Item 1, ‘being listened to’, and Item 6, ‘being informed about what is done’. The attributes with the highest number of somewhat important answers were Items 2, 8 and 9: ‘my experiences being recognised’, ‘taking part in planning’ and ‘phrasing personal goals’ (the latter indicating the largest variation across the response alternatives for Item 9). The least‐preferred attributes for their participation were ‘managing treatment’ (Item 11) and ‘managing self‐care’ (Item 12), as shown in Figure 2A.

Regarding patient experiences of participation, between 15% and 23% reported that they had entirely experienced conditions for their participation. Most patients reported that they had, to a large extent, experienced conditions for ‘being listened to’ (Item 1) and ‘sharing my symptoms’ (Item 4). Altogether, for five items, most patients reported having partaken in no or only to some extent conditions: Items 5, 8, 9, 10 and 11 (i.e., having explanations of symptoms, taking part in planning, phrasing personal goals, learning to manage symptoms, and managing treatment myself). All details of patients' experiences with patient participation are provided in Figure 2B for more information.

#### 3.2.1 Preference‐based patient participation

Frequent instances of agreement were observed when comparing patients' actual experiences with patient participation to their expressed preferences for such engagement. Between 20% and 45% of the patients completely matched their preferences for and experiences with participation. Meanwhile, for more than half of the items, more than half of the respondents showed a sufficient match between preferences and experiences: Items 2, 4, 6, 8, 9, 11 and 12 (i.e., one's experiences being recognised, sharing one's symptoms, being informed of what has been done, taking part in planning, phrasing goals, managing treatment and managing self‐care).

**Figure 3:**
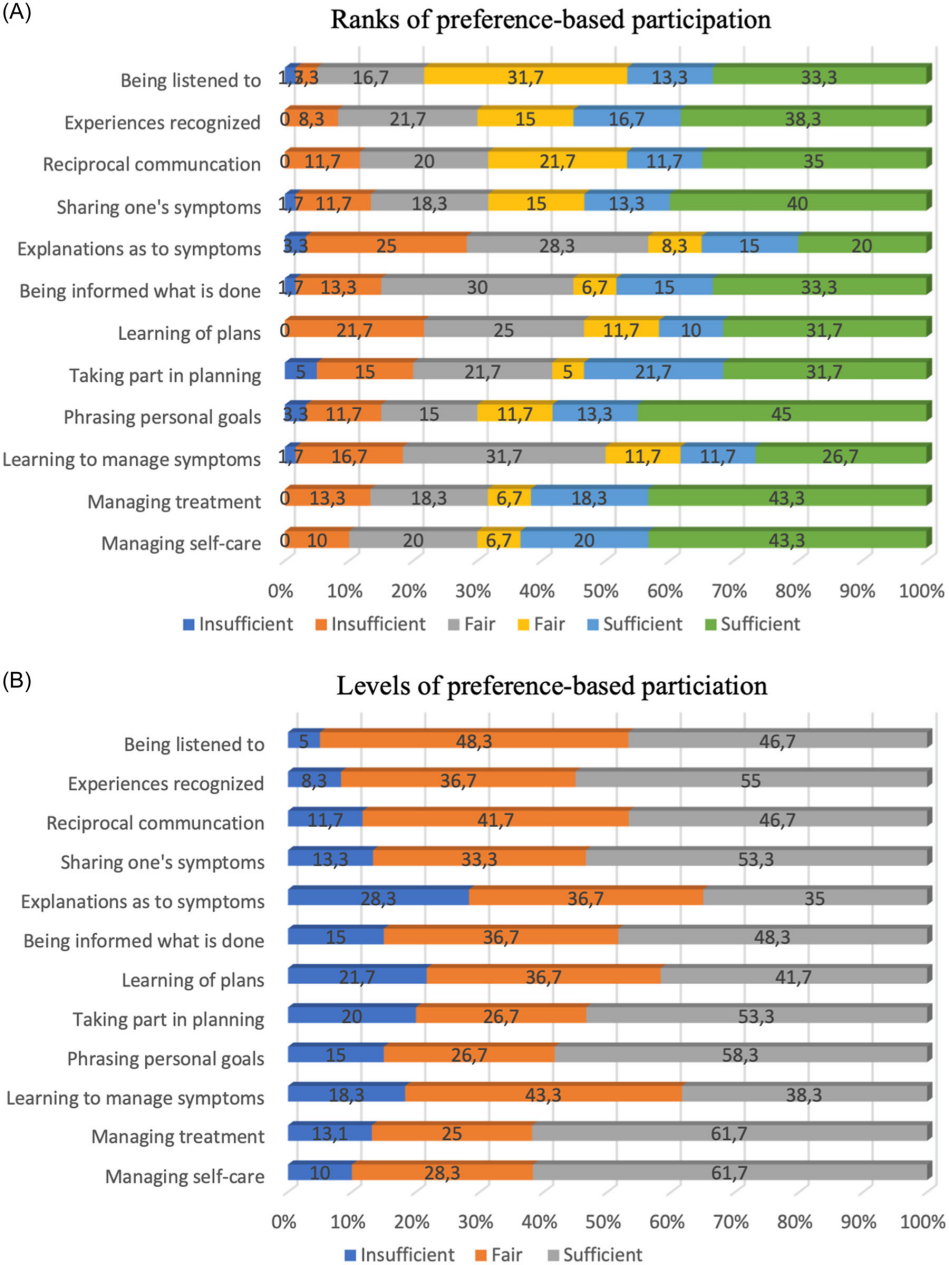
The percentage of preference‐based patient participation per item was assessed using ranks (A) and levels (B). Ranks 0–1 indicated an insufficient level, Ranks 2–3 indicated a fair level, and ranks 4–5 indicated a sufficient level of patient participation.

Besides the approximately 50% of the respondents reaching a sufficient level (i.e., ranks 4 and 5) of preference‐based patient participation in the IC context, there were higher levels of mismatches for Items 1, 3, 5, 7 and 10: for ‘being listened to’, ‘having reciprocal communication’, ‘having explanations for one's symptoms’, ‘learning of plans’ and ‘learning to manage symptoms’. The most mismatches for preference‐based patient participation were for Items 5 and 10, including ‘having explanations of one's symptoms’ and ‘learning to manage symptoms’. All details are presented in Figure 3.

**Figure 4:**
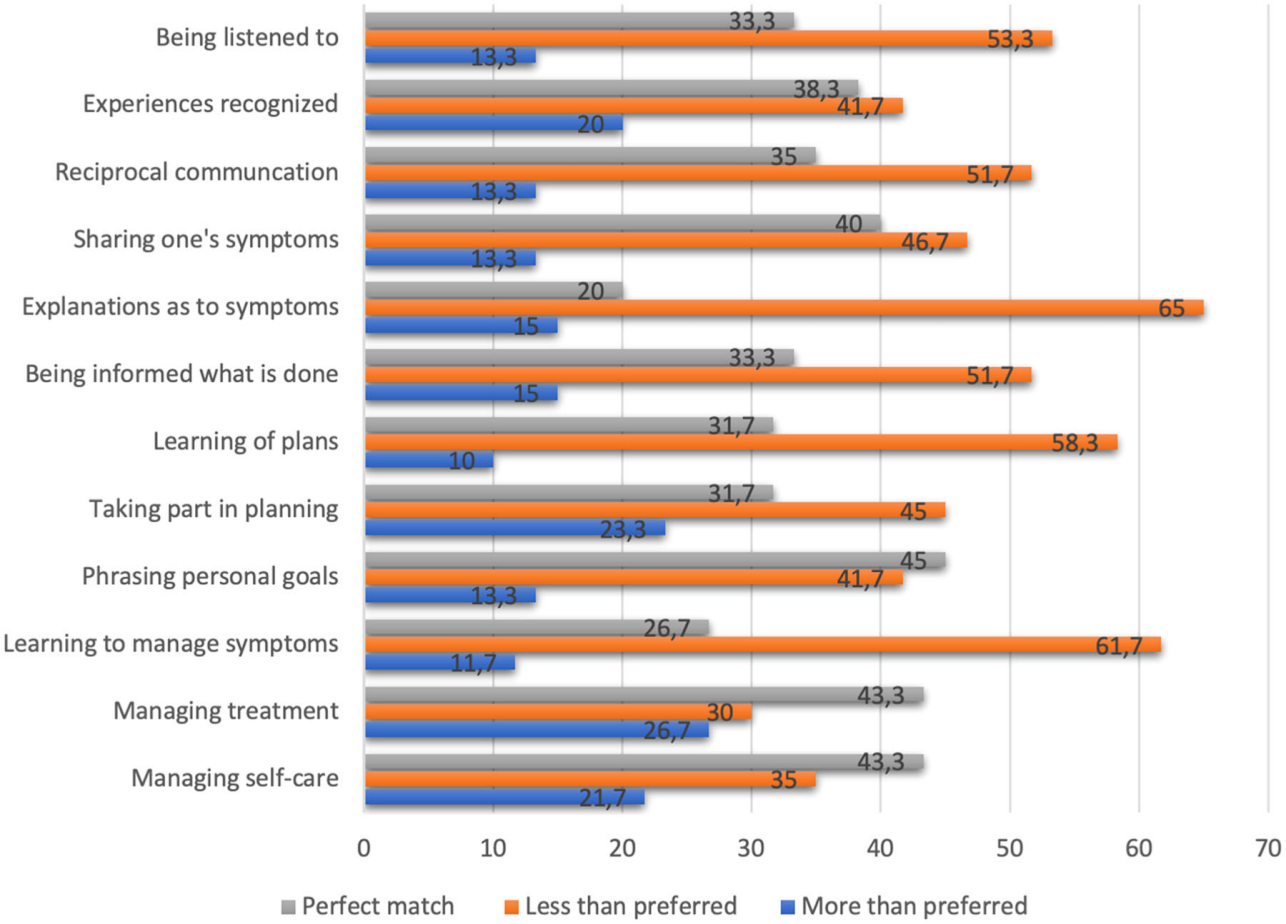
Proportions of matches (%) indicating whether the respondents experienced conditions exceeding the participation preferred, reduced participation compared to the preferred or a match between preferences and experiences.

Another glance at the mismatches between the patients' preferences for and experiences with participation indicates that these most often implied lesser conditions than patients preferred. This was most evident for the items ‘having an explanation of symptoms’ (65%), ‘learning to manage symptoms’ (61.7%) and ‘learning of plans’ (58.3%). The respondents experienced more conditions than they preferred for their participation for the item ‘managing treatment’ (26.7%). Furthermore, the item ‘phrasing personal goals’ had the highest proportion of complete matches (45%). Simultaneously, a large proportion of the participants experienced receiving less patient participation than they preferred (41.7%). All details regarding the proportions of matches are provided in Figure 4.

## 4 DISCUSSION

To the best of our knowledge, there are no prior PREM evaluating preference‐based patient participation validated for the Norwegian IC context. Rather, in this study, we conducted a careful translation, content validation and initial piloting of the 4Ps instrument with patients in Norwegian IC services. The main findings of this study reflect that the translation between Swedish and Norwegian was straightforward and required no conceptual or contextual adaptations, as further discussed. The subsequent cross‐sectional study, designed as a dialogue between patients and HCPs, revealed that only 50% of individuals reached a sufficient level of patient participation, mostly experienced as receiving less‐than‐preferred conditions for engaging in one's health and healthcare issues. The latter corresponds well with previous research on transitional care, highlighting that older patients experience a lack of participation.^26^, ^43^

Overall, the translated version was equivalent to the original version, with only minor discussions, mostly related to the use of synonyms. An explanation might be that Norwegian and Swedish are closely related, both linguistically and culturally. Moreover, the legitimacy of the original version was prioritised since high‐quality research requires access to valid and reliable instruments that can be used across cultures and languages.^39^ Even with the guidelines available for translating and validating patient‐reported outcome measures, there is a diversity in methodological approaches^38^ and a lack of consensus on how to use and/or combine the steps. For example, a common strategy is only to report the forward translation, which is often conducted by an unqualified translator.^37^ However, in this study, we incorporated a multistep process, using both qualitative and quantitative methods (in accordance with contemporary guidelines) for trustworthiness.^36^

The respondents interviewed found the 4Ps relevant, understandable and user friendly, rather similar to the original (i.e., Swedish) findings.^29^ Nonetheless, the context was separate in our study, with an expected level of frailty among patients admitted for IC services, corroborating previous studies on transitional care.^43^ To facilitate individual responses, Section 1 of the 4Ps was read aloud: The patient's preferences for participation were mapped within the first days after admission, while Section 2 evaluated their experiences during the final days of their stay. While this also provides HCPs with an opportunity to evaluate the conditions for preference‐based patient participation,^29^ current studies indicate that staff and managers may need further support for implementing such internal quality assessments. However, while previous studies employing the 4Ps have solely used self‐reports filed by researchers,^31^, ^44^, ^45^, ^46^ we propose future studies to further engage in a mutual coproduction of patient participation.^47^

Patient participation entails that the provision of care should be guided by the patients' needs, goals and conditions and should include opportunities for participation.^20^ However, our results from the cross‐sectional study revealed that 50% of the subjects received a sufficient level of patient participation based on their preferences. Overall, this is a lower level than some previous studies of the 4Ps, such as surgical cancer care^45^ and renal care,^46^ where approximately two‐thirds reached a sufficient level. However, the findings correspond well with previous research on IC,^23^, ^24^, ^25^ indicating room for more person‐centred patient participation. Most importantly, in all items, our results revealed that experienced patient participation was less than preferred. A recent study in kidney care, where patients tend to be the same age, indicated similar findings: Patients are deprived of engagement opportunities for most items. However, when it comes to performing prescribed care, there is a tendency for the HCP to expect more patient participation than the patient is prepared for.^46^

What patients prioritise for their participation in IC is ‘being listened to’ (Item 1) and ‘having reciprocal communication’ (Item 3). Drott et al.^45^ also found that this was the case for surgical cancer patients, indicating the importance of constructive dialogues with HCPs. Any such dialogue should be two‐way to provide a mutual exchange of information and knowledge, including opportunities to address barriers such as reduced health literacy and/or fatigue.^48^ This also facilitates engagement in the further planning of one's care and in managing self‐care.

With IC patients' preferences not matching their experiences (but the mismatches most often implying lesser conditions than preferred), increased engagement is particularly needed in symptom management (items ‘having explanation of symptoms’ and ‘learning to manage symptoms’). Today, due to increased needs and a lack of resources, even older persons face early discharge from the hospital, often in a still‐frail condition. Nevertheless, the context of IC is supposed to deliver rehabilitation to facilitate an efficient flow from the hospital to the home through a person‐centred process.^11^ However, there is tension between the standardisation and individualisation of the care provided during transitions from hospital to home for older patients, jeopardising the quality of their healthcare experience. HCPs need room in their everyday practice for professional flexibility to meet patients' individual needs while ensuring evidence‐based practice.^43^

One such aspect is highlighted by the 4Ps item ‘phrasing personal goals’. In this study, it showed the largest variation across the response alternatives. Goal setting is a core practice within rehabilitation (and in family meetings in IC) deemed vital to motivate the patient for their rehabilitation and to increase behaviour change.^49^ We suggest continuing the interdisciplinary work within teams, with patients and their next‐of‐kin, initiated by the query ‘What is important to you?’ within IC services.^25^ Asking ‘What is important to you?’ has been promoted for the implementation of person‐centred care.^50^ Based on the concept of person‐centred care, focusing on the elements of care, support and treatment that hold the utmost importance to the patient and their next of kin,^21^ our findings revealed that individuals too seldom experienced person‐centred care. Nonetheless, this approach is recognised for its ability to yield benefits not only in terms of improved health outcomes and patient satisfaction but also in terms of reduced healthcare costs.^51^ Although the question ‘What is important to you?’ seems simple, using the question requires professional competence: An important factor is for HCPs to have and perceive the relevant limits of their provision of care (while the broader context of the patient's life project may merit additional resources beyond healthcare services).^52^

Our findings might encourage a reflection on what competences HCPs currently use and need when engaging with chronically ill people with respect to patient participation. HCPs, including leaders and organisations, must make room for reflection on the conditions staff have for engaging with patients during their IC stays. For example, interprofessional simulation‐based education can improve both teamwork and HCPs' communication skills^53^, ^54^ and we suggest that the Norwegian 4Ps may serve as a route to grasp what is important for IC patients' person‐centred participation.

### 4.1 Strengths and limitations of this study

To establish trustworthiness, the multistep process conducted in this study incorporated both qualitative and quantitative methods (i.e., triangulation).^39^ The project team incorporated relevant lexical, conceptual and contextual experiences for the translations and cross‐cultural adaptations. In addition, we enroled two independent bilingual back translators who were blinded to the original version. Overall, this procured a detailed translation and verified the credibility of the study.^36^

Face‐to‐face interviews were time‐consuming and resource intensive with the patients admitted to IC and HCPs in addressing the relevance, comprehensiveness and clarity of the 4P instrument. However, we trust they served the Norwegian version of the 4Ps. While all feedback from the interviewees was noted immediately in a summarising memo after every interview, the 4Ps dialogues were not audiotaped. Consequently, nuances in the participants' feedback may have gone missing.

Despite the protocol, interviews are known to be subject to participants' reporting bias.^55^ Although the HCPs were not interviewing the patients they oversaw as professionals, patients may have found it difficult to report negative experiences during their stay. We do not report differences in responses based on length of stay due to missing data on this issue. Rather, this might be explored further in a larger sample in future research. Finally, due to the small sample size in this cross‐sectional study, the findings should be carefully interpreted and presumably explored in larger settings and samples.

## 5 CONCLUSION

This study represents a careful translation, a content validation and a first piloting of the 4Ps instrument with patients in IC services in Norway. The translation process was easy, with few deviations. Both questions and response options were understood by the respondents and seemed relevant in measuring patient participation. The cross‐sectional study, designed as a dialogue between the patients and HCPs, revealed that only 50% of the interviewees had a sufficient level of preference‐based patient participation, mostly represented as having fewer conditions for their participation than they preferred. Hence, a more empowering, person‐centred way of ensuring patient participation in IC is expected.

The results of our study can facilitate nuanced discussions on the topic, guide targeted research within the field and enhance interventions in the context of IC, particularly with regard to preference‐based patient participation. To instigate such quality improvement, an awareness of the barriers and facilitators of one's service unit is helpful. The 4Ps instrument seems suitable for measuring patient participation based on patient preferences in the IC context and is feasible for both HCPs and patients to complete in an interview when arriving at and leaving services. This may support person‐centred communication and collaboration, calling for further research on what facilitates patient participation and the implementation of person‐centred services for patients in the IC context.

## CONFLICT OF INTEREST STATEMENT

The authors declare no conflict of interest.

## Supporting information

Supporting information.

Supporting information.

Supporting information.

## Data Availability

The data that support the findings of this study are available from the corresponding author upon reasonable request.
