# Supplementary material for: Preference‐based patient participation in intermediate care: Translation, validation and piloting of the 4Ps in Norway
Source: Health Expect. 2023 Nov 7;27(1):e13899. doi: 10.1111/hex.13899 (PMC10726279; doi:10.1111/hex.13899)
Supplement: Supplementary file 1 — Supporting information. [file HEX-27-e13899-s001.docx]

**Interview guide the 4Ps**

The cognitive interviews are individual and face-to-face, and will be carried out to evaluate the instructions, response format, and the 4P items and response alternatives. During the interviews, the informants will be asked to fill out the 4Ps, while simultaneously thinking aloud, addressing the relevance, comprehensiveness, and clarity of the 4Ps. The researcher will subsequently write down the informants' feedback. The information will be anonymized.

**Relevance**

1. Do you feel the included items are relevant for assessing patient participation?
2. Do you find the included items relevant and appropriate to ask older people?
3. Do you experience the included items as relevant for the intermediate care context?
4. Do you find the response options within the 4Ps as relevant and appropriate?
5. Do you find the time for the completion of both versions as appropriate?

**Comprehensiveness**

1. Do you feel that all the key dimensions for assessing patient participation are included?

**Comprehensibility**

1. Do you find the instructions simple and understandable?
2. Do you feel that the various items and response options are logic and understandable?
3. Do you think the items are appropriately worded?
4. Do you feel that the response options match the questions in the instrument?
