# Supplementary material for: Preference‐based patient participation in intermediate care: Translation, validation and piloting of the 4Ps in Norway
Source: Health Expect. 2023 Nov 7;27(1):e13899. doi: 10.1111/hex.13899 (PMC10726279; doi:10.1111/hex.13899)
Supplement: Supplementary file 2 — Supporting information. [file HEX-27-e13899-s003.docx]

**Preference-based patient participation (The 4Ps)**

**Purpose of the test protocol**

This test protocol has been developed to ensure standard procedure when carrying out the 4Ps as part of the IPIC study (Implementation of Patient participation in Intermediate Care). The protocol is based on the Norwegian translation by Kvæl, Bergland and Eldh (2022).

| Overview | The 4Ps consists of two sections and sports 12 items. The first section of the 4Ps, i.e., the patient’s preferences for patient participation, will be completed within the first days after the patient’s admission. Section two, i.e., the patient’s experience of patient participation, will be completed during any of the final days before the discharge to their home. The two sections are then compared, evaluating whether there is a fit between the individuals’ preferences and experiences for patient participation. |
| --- | --- |
| Guidelines | 1. The test is carried out in collaboration between the patient and an healthcare professional (HCP) 2. The same HCP performs both sections for the respective patient 3. The HCP carries out the test in line with the procedure described below 4. Avoid any discussions related to the specific items during the process 5. Find a suitable time appropriate for the patient to conduct the 4Ps 6. The HCP should not have any treatment relationship to the patient 7. Part 1 should be completed within the 3^rd^ working day after admission 8. Part 2 should be completed during the last two days before discharge 9. Two HCPs from each IC will be responsible for the data collection 10. All data collectors will undergo systematic and 'equal' training |
| Equipment | You will need a paper version of the 4P test and a pen. Sitting around a table, for example in the patient room. |
| Time | Up to 15 minutes for each section. |
| Data collector | Complete the scoring sheet based on the patient's answers to the 12 items in the given order. Avoid looking at section 1 when completing section 2. |
| Introduction | Obtain informed consent (signature) after giving oral and written information about the project. |
| Instruction | - See standardized instructions below for each part. In case of cognitive difficulties, the instruction can be repeated to ensure that the patient has understood the aspect of participation or the various response options. - Item 11: ‘managing treatment myself’ with the subsequent example of ‘manage my medication’. If necessary, add the example of ‘compliance to exercise’ as this may be relevant for the intermediate care context. - Item 12: ‘managing self-care’ with the subsequent example of ‘adapting my diet’. If necessary, add the example of ‘being independent in my ADL’ as this may be relevant for the intermediate care context. |
| Finalizing/  Summing up | - «Thank you for your time and for sharing your preferences and experiences of patient particiation. Your information will not be shared with any employees in IC but will solely be part of this research project. The results will help us understand how we better can support person-centred communication and collaboration in the context of intermediate care. If you have any questions about the study, please contact the researcher stated in the information letter» (please ensure the patient has the information letter or provide a new one if necessary). - Please remember to fill in the background information sheet. |

Standard procedure section 1:

Read the instruction on page 1 out loud for the patient:

«*All health care should be based on you, as a patient, having conditions for participation*.  *...*»

Once you have read the instruction, explain how you will aid the patient:

*«Now, I will assist you filling in the first section of the 4Ps, i.e., your preferences for patient particiation. I will read for you 12 aspects of patient participation. Please rate each aspect as either «not important», «somewhat important”, “very important” or “crucial” for you to experience particiation while being a patient in IC. Based on your current situation, what is important to you?”* (repeat if necessary).

1. For me, to experience patient participation it is «not important», «somewhat important», «very important» or «crucial» that healthcare staff listen to me
2. For me, to experience patient participation it is «not important», «somewhat important», «very important» or «crucial» that my experiences are recognized
3. For me, to experience patient participation it is «not important», «somewhat important», «very important» or «crucial» that there are conditions for reciprocal communication
4. For me, to experience patient participation it is «not important», «somewhat important», «very important» or «crucial» having conditions for sharing my symptoms/issues
5. For me, to experience patient participation it is «not important», «somewhat important», «very important» or «crucial» having explanations of my symptoms/issues
6. For me, to experience patient participation it is «not important», «somewhat important», «very important» or «crucial» that the healthcare staff explain what will be/is done for me
7. For me, to experience patient participation it is «not important», «somewhat important», «very important» or «crucial» that I learn what is planned for me
8. For me, to experience patient participation it is «not important», «somewhat important», «very important» or «crucial» having conditions for being involved in planning of care/treatment
9. For me, to experience patient participation it is «not important», «somewhat important», «very important» or «crucial» having conditions for phrasing personal goals
10. For me, to experience patient participation it is «not important», «somewhat important», «very important» or «crucial» having conditions to know how to manage with my symptoms/issues
11. For me, to experience patient participation it is «not important», «somewhat important», «very important» or «crucial» having conditions for managing treatment, myself, such as my medication (NB: this is about prescribed treatment, in IC care this could for example be ‘compliance to exercise’)
12. For me, to experience patient participation it is «not important», «somewhat important», «very important» or «crucial» having the conditions for managing self-care, such as adapting my diet (add, if necessary, the example ‘being independent in my ADL’)

Standard procedure section 2:

*«When you arrived, I helped you fill in part 1 of the 4Ps, which embraced your preferences for patient participation. I will now assist you filling in part 2 of the 4Ps. Here, you are asked to indicate the extent to which you have experienced the same aspects of participation while you have been a patient in IC. I want to emphasize that what you have experienced is right for you, there are no wrong answers. As before in section one, I will read the 12 aspects of participation. Please assess the extent to which you have experienced the various aspects of patient particiation. The answer options are: "not at all", "to some extent", "to a large extent" or "entirely"»* (repeat if necessary).

1. The healthcare staff have listened to me: «not at all», «to some extent», «to a large extent» or «entirely»
2. My experiences have been recognized: «not at all», «to some extent», «to a large extent» or «entirely»
3. There have been conditions for reciprocal communication: «not at all», «to some extent», «to a large extent» or «entirely»
4. I have had conditions to tell about my symptoms/issues: not at all», «to some extent», «to a large extent» or «entirely»
5. I have had explanations as to my symptoms/issues: «not at all», «to some extent», «to a large extent» or «entirely»
6. Healthcare staff have explained what will be/is done for me: «not at all», «to some extent», «to a large extent» or «entirely»
7. I have learned what is planned for me: «not at all», «to some extent», «to a large extent» or «entirely»
8. I have had conditions to take part in planning care/treatment: «not at all», «to some extent», «to a large extent» or «entirely»
9. I have had conditions for phrasing personal goals: «not at all», «to some extent», «to a large extent» or «entirely»
10. I have had conditions to learn how to manage my symptoms/issues: «not at all», «to some extent», «to a large extent» or «entirely»
11. I have had conditions to manage treatment myself, such as to manage my medication: «not at all», «to some extent», «to a large extent» or «entirely» (if necessary, add the example ‘compliance to exercise’)
12. I have had conditions to manage self-care, such as adapting my diet: «not at all», «to some extent», «to a large extent» or «entirely» (if necessary, add the example ‘being independent in my ADL’)
