# Supplementary material for: Preference‐based patient participation in intermediate care: Translation, validation and piloting of the 4Ps in Norway
Source: Health Expect. 2023 Nov 7;27(1):e13899. doi: 10.1111/hex.13899 (PMC10726279; doi:10.1111/hex.13899)
Supplement: Supplementary file 3 — Supporting information. [file HEX-27-e13899-s002.pdf]

4P – pasientdelaktighet ut fra pasientpreferanser

Helsevesenet skal baseres på at du, som pasient, gis forutsetninger for å være delaktig i omsorg og behandling. Å være delaktig kan bety ulike ting for ulike personer ved ulike tilfeller, avhengig av ens sykdom eller livssituasjon.

Dette spørreskjemaet undersøker hvilke aspekter du, som pasient, prioriterer i spørsmålet om delaktighet **og** i hvilken utstrekning du har opplevd delaktighet i din kontakt med helsevesenet.

I skjemaet presenteres en rekke aspekter ved pasientdelaktighet. Disse er basert på studier av hva personer i pasientrollen beskriver som pasientdelaktighet, lovtekster og andre styrende dokumenter i helsevesenet samt ordbøker og annen litteratur.

I den første delen angir du for hvert enkelt aspekt hvor viktig det er for at du skal oppleve delaktighet. Ta utgangspunkt i din aktuelle situasjon og kontakt med helsevesenet. Kryss av ett alternativ for hvert enkelt av delaktighetsaspektene.

I den andre delen angir du i hvilken utstrekning du har opplevd delaktighet i din kontakt med helsevesenet. Som det fremgår av instruksjonen ber vi deg også her å krysse av ett alternativ for hvert enkelt av delaktighetsaspektene. Også i denne delen ta utgangspunkt i din aktuelle situasjon og omsorgskontakt.

© Ann Catrine Eldh og Linda Aimée Hartford Kvæl
Får ikke kopieres, endres eller distribueres uten tillatelse.

For at jeg skal oppleve delaktighet er det:

	ikke viktig	ganske viktig	veldig viktig	avgjøren-de
1. at helsepersonell lytter til meg	☐	☐	☐	☐
2. at mine erfaringer ivaretas	☐	☐	☐	☐
3. at det finnes forutsetninger for gjensidig kommunikasjon	☐	☐	☐	☐
4. at jeg får forutsetninger for å fortelle om mine symptomer/plager	☐	☐	☐	☐
5. at jeg får forklaring på mine symptomer/plager	☐	☐	☐	☐
6. at helsepersonell forklarer hva som skal gjøres/gjøres for meg	☐	☐	☐	☐
7. at jeg får vite hva som planlegges for meg	☐	☐	☐	☐
8. at jeg får forutsetninger for å være med i planlegging av omsorg/behandling	☐	☐	☐	☐
9. at jeg får forutsetninger for å sette egne mål	☐	☐	☐	☐
10. at jeg får forutsetninger for å vite hvordan jeg skal gjøre for å håndtere mine symptomer/plager	☐	☐	☐	☐
11. at jeg får forutsetninger for å håndtere behandling selv, som for eks. å ordne med egne medisiner	☐	☐	☐	☐
12. at jeg får forutsetninger for egenomsorg, som for eks. å tilpasse kosten	☐	☐	☐	☐

© Ann Catrine Eldh og Linda Aimée Hartford Kvæl
Får ikke kopieres, endres eller distribueres uten tillatelse.

På forrige side ble du bedt om å angi hvor viktig ulike aspekter ved delaktighet er for deg i din omsorgskontakt. Her bes du i stedet **angi i hvilken utstrekning du har opplevd ulike aspekter ved delaktighet**. Ta utgangspunkt i din aktuelle omsorgskontakt. Kryss av ett av de foreslåtte alternativ for hvert enkelt av delaktighetsaspektene.

	**Ikke i det hele tatt**	**Til en viss grad**	**I stor grad**	**Fullsten-dig**
1. Helsepersonell har lyttet til meg	☐	☐	☐	☐
2. Mine erfaringer har blitt ivaretatt	☐	☐	☐	☐
3. Det har vært forutsetninger for gjensidig kommunikasjon	☐	☐	☐	☐
4. Jeg har fått forutsetninger for å fortelle om mine symptomer/plager	☐	☐	☐	☐
5. Jeg har fått forklaring på mine symptomer/plager	☐	☐	☐	☐
6. Helsepersonell har forklart hva som skal gjøres/gjøres for meg	☐	☐	☐	☐
7. Jeg har fått vite hva som planlegges for meg	☐	☐	☐	☐
8. Jeg har fått forutsetninger for å være med i planlegging av omsorg/behandling	☐	☐	☐	☐
9. Jeg har fått forutsetninger for å sette egne mål	☐	☐	☐	☐
10. Jeg har fått forutsetninger for å vite hvordan jeg skal gjøre for å håndtere mine symptomer/plager	☐	☐	☐	☐
11. Jeg har fått forutsetninger for å håndtere behandling selv, som for eks. å ordne med egne medisiner	☐	☐	☐	☐
12. Jeg har fått forutsetninger for egenomsorg, som for eks. å tilpasse kosten	☐	☐	☐	☐

© Ann Catrine Eldh og Linda Aimée Hartford Kvæl
Får ikke kopieres, endres eller distribueres uten tillatelse.
